# Fluorescence Sensors for the Detection of L-Histidine Based on Silver Nanoclusters Modulated by Copper Ions

**DOI:** 10.3390/molecules29102167

**Published:** 2024-05-07

**Authors:** Yuxia Li, Min Li, Liuzhi Hu, Baozhu Zhang

**Affiliations:** Department of Chemistry and Chemical Engineering, Jinzhong University, Jinzhong 030619, China; yxli_2004@163.com (Y.L.); lm19834731959@outlook.com (M.L.); qijiaoban2023@163.com (L.H.)

**Keywords:** fluorescence sensors, silver nanoclusters, L-histidine, copper ions

## Abstract

In this study, Cu^2+^ modulated silver nanoclusters were constructed for the turn-on, label-free detection of L-histidine. Six Ag NCs protected by oligonucleotides (DNA-Ag NCs) were tested in a series of experiments. Finally, A-DAN-Ag NCs were chosen as the best candidate due to their excellent fluorescent properties. The fluorescence of A-DAN-Ag NCs was quenched using Cu^2+^ through energy or electron transfer. However, quenched fluorescence could be restored dramatically in the presence of L-histidine due to Cu^2+^ liberation from A-DAN-Ag NCs and because of the chelation between the imidazole group of L-histidine and Cu^2+^. The proposed sensor exhibited high selectivity towards L-histidine over other amino acids, with a limit of detection (LOD) of 0.096 μM ranging from 0 to 8 μM. The proposed sensor succeeded in detecting L-histidine in diluted human urine. Therefore, the sensor has promising practical applications in biological systems.

## 1. Introduction

In recent years, fluorescent mental nanoclusters (NCs) consisting of several to hundreds of atoms have attracted considerable interest because they are less than 2 nm in size, comparable to the Fermi wavelength of electrons, and display molecule-like properties such as discrete energy levels and luminescence [1]. Compared with quantum dots (QDs) and small-molecule dyes, they have many advantages including ultrasmall size (<2 nm), larger Stokes shift, good photostability, low toxicity, easy synthesis [2,3], etc. Among all types of metal nanoclusters, silver nanoclusters stabilized by oligonucleotides or single-stranded DNA exhibit good photophysical and spectral properties; for example, the fluorescence emission bands of DNA-Ag NCs can be adjusted from the visible to near-IR region via different DNA secondary structures and sequences [4,5]. On the other hand, they possess good biocompatibility and water solubility and facilitate bioconjugation [6]. Thus, various analytes were detected using DNA-Ag NCs, including small molecules [7,8]. metal ions [9,10,11,12], DNA [13,14,15], enzymes [16,17,18], proteins [19,20,21], etc.

L-histidine exists in the protein of humans and other mammals as an essential amino acid. The aromatic nitrogen–heterocyclic imidazole side chain of L-histidine can control metal transport in biologically important bases [22] and minimize internal bleeding due to microtrauma [23]. It has been reported that chronic kidney disease [24], the failure of normal erythropoiesis development, Parkinson’s disease, and Friedreich ataxia [25], are caused by deficiency in L-histidine. In addition, histidine mia or metabolic disorders can occur if L-histidine levels are higher than normal in physiological fluids including urine and serum [26]. In recent years, several methods detecting L-histidine have been developed, such as electrochemistry [27], capillary electrophoresis [28], colorimetry [29,30], liquid chromatography [31], and spectro fluorimetry [32,33,34,35]. Although some of these methods succeed in sensitivity, selectivity, and detection limit, most require expensive robotic facilities, heavy optical equipment, complicated operation procedures, and a lower detection limit; moreover, the biological sample requires time-consuming pre-treatment. Among the abovementioned methods, fluorescent biosensors possess the advantages of high sensitivity, simple operation procedures, long linear range, low detection limit, etc. Despite the fact that fluorescent methods have made significant progress in the detection of L-histidine, they are still limited in utilizing quantum dots and small-molecule dyes as probes. These probes have disadvantages including challenging synthesis, high toxicity, and low photostability [30,36,37]. Hence, developing low-cost, highly selective fluorescence sensors for detecting L-histidine is still necessary to overcome the abovementioned drawback.

Until now, among the fluorescent sensors, sensors based on DNA-Ag NCs for detecting L-histidine may overcome the inherent drawbacks of quantum dots and small-molecule dyes. Song et al. have developed a strategy for the detection of histidine based on gold nanoclusters complexes modified by Ni^2+^, with a limit of detection of 30 nM in the linear range of 0.1–26 μM [32]. Yao et al. have also explored a probe for the detection of histidine based on silver nanoclusters modified by Cu^2+^, with a limit of detection of 4.3 nM in the range of 0.20–80 μM [38]. The latter precedes the former with respect to the linear range and LOD. However, their performances are still unsatisfactory. Therefore, exploring highly selective and sensitive simple operational methods is urgently needed for L-histidine quantitative analysis.

**Scheme 1:**
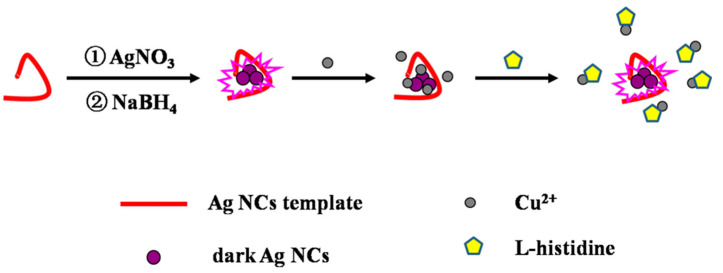
Schematic illustration of the fluorescence sensor for the detection of L-histidine with A-DNA-Ag NCs.

Herein, a turn-on fluorescence sensor was constructed based on DNA-Ag NCs modulated by copper ions for the detection of L-histidine. It has been reported that DNA-Ag NCs protected by different DNA templates possess different optical properties, which depend on the characteristics of their secondary structures and base sequences [39,40]. The properties (for example, excitation and emission wavelength) of Ag NC DNA templates containing different secondary structures, such as G-quadruplex and the i-motif, may vary due to Ag NCs being in different microenvironments. Hence, six different sequence DNA strands were experimentally analyzed, and the best (A-DNA-Ag NCs) one was chosen due to its excellent fluorescence properties. As indicated in Scheme 1, fluorescence quenching in A-DNA-Ag NCs is a result of the transfer of energy or electrons via Cu^2+^. The fluorescence was restored in the presence of L-histidine due to the coordination of the imidazole group of histidine with Cu^2+^ results in Cu^2+^ liberation from DNA-Ag NCs [38]. To validate Scheme 1, the fluorescent spectra of A-DNA-Ag NCs in different conditions were measured (SI Figure S1). It was observed that A-DNA-Ag NCs exhibited stronger fluorescence (curve a) and, then became weak (curve b) in the presence of 400 nM Cu^2+^; finally, fluorescence increased significantly (curve c) upon adding 10 mM of L-histidine. Therefore, the scheme is feasible. As a result, it enabled the convenient, selective, and turn-on fluorescence detection of L-histidine and successfully detected L-histidine in diluted human urine samples. Therefore, its application in biological and clinical diagnosis fields exhibits great potential.

## 2. Result and Discussion

### 2.1. Optical Characterization of DNA-Ag NCs

**Figure 1:**
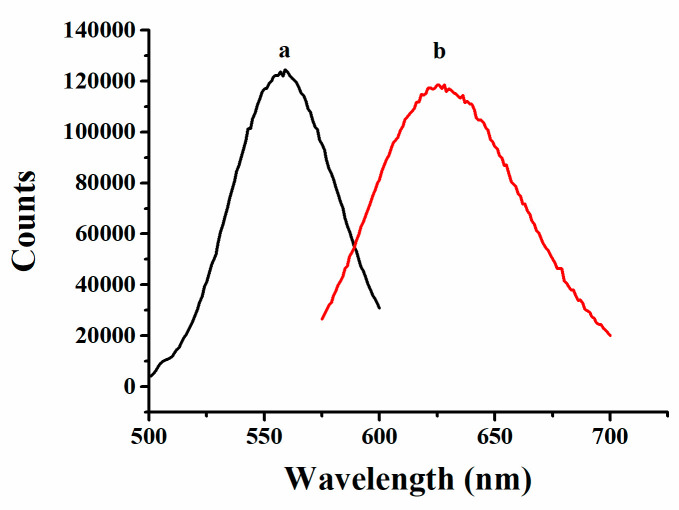
The excitation (a) and emission (b) spectra of A-DNA-Ag NCs.

The base sequences and secondary structures of DNA templates play a key role with respect to determining the optical properties of metal NCs [39,40]. Since different DNA templates possess different sequences and second structures, resulting in DNA-Ag NCs with different compositions and structures, their properties are also different with respect to excitation wavelengths, emission wavelengths, etc. Therefore, six DNA templates named A, B, C, D, E, and F-DNA were chosen because they can be found in the most commonly used nucleation sequences [41,42]. Table 1 lists their sequences and names. They all contain C bases. B, C, E, and F-DNA possess T and A bases in addition to C bases; D-DNA possesses T bases in addition to C bases; and A-DNA only possesses C bases. As shown in Figure 1, the excitation and emission spectra of A-DNA-Ag NCs are represented by curve a and b, and the excitation and emission peaks are 555 nm and 625 nm, respectively. Similarly, the excitation and emission spectra of B, C, D, E, and F-DNA-Ag NCs are shown in SI Figure S2. Their excitation and emission wavelengths are 595 nm, 630 nm, 555 nm, 575 nm, 566 nm, and 675 nm, 710 nm, 630 nm, 655 nm, and 645 nm, respectively. The UV–Vis absorption spectra of A-DNA-Ag NCs were measured upon the addition of different concentrations of L-histidine. SI Figure S3 displays an obvious peak (430 nm) in each absorption spectrum. The peak at 430 nm can be attributed to the characteristic plasmon absorption band of Ag nanoparticles [43]. The absorption intensity becomes stronger and stronger with increasing concentrations of L-histidine. Thereby, A-DNA-Ag NCs also contain Ag nanoparticles. However, the absorption spectra of A-DNA-Ag NCs are not obvious due to the insufficient amount of A-DNA-Ag NCs.

The fluorescence intensity of six DNA-Ag NCs against storage time was measured due to the substantial effect on the detection performance for the stability of the sensor. As displayed in the SI shown in Figure S4, the fluorescence intensity of A-DNA-Ag NCs reached a plateau after 1 h, and remained stable for 1.5 h, gradually declining thereafter. There was a similar trend of change in the fluorescence intensity of B and C-DNA-Ag NCs. Their fluorescence intensity was gradually enhanced at the beginning and subsequently remained unchanged. The fluorescence intensity of D-DNA-Ag NCs reached a maximum in a short period of time and then gradually decreased finally stabilizing. However, the fluorescence intensity of E and F-DNA-Ag NCs decreased at first and gradually became stable. Although A-DNA-Ag NCs can maintain stability for only 1.5 h, they need to be improved in order to expand their applicability in the future. Taking other optimization conditions into account, A-DNA-Ag NC was finally selected as the probe for detecting L-histidine.

The absolute photoluminescence quantum yields of A, B, C, D, E, F-DNA-Ag NCs were measured for the evaluation of fluorescence emissions. As displayed in SI Figure S5, they are 36.51%, 14.98%, 9.22%, 8.81%, 12.28%, and 4.06%, respectively. It is apparent that the maximum quantum yield of A-DNA-Ag NCs is consistent with the highest number of Ag NC nucleation sequences.

### 2.2. Characteristics of A-DNA-Ag NCs

**Figure 2:**
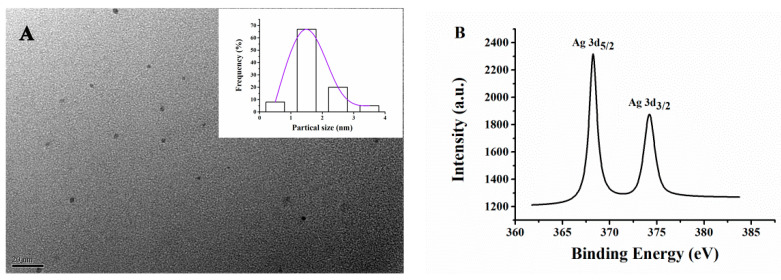
(**A**) TEM image of A-DNA-Ag NCs (inset: the size distribution histogram). (**B**) XPS spectrum of Ag 3d for A-DNA-Ag NCs.

TEM was performed to characterize the morphology and size of A-DNA-Ag NCs. As shown in Figure 2A, the average diameter of the A-DNA-Ag NCs was about 2 nm, consistent with the size of metal NCs being less than 2 nm [44], and this was observed in a histogram fitted utilizing the Lorentzian function. On the other hand, TEM also shows that the sample is a mixture of Ag NPs and nanoclusters of different sizes.

In order to illustrate that the Ag elements of A-DNA-Ag NCs exist in a valence state, the XPS spectrum of Ag 3d was examined. Two peaks are displayed in Figure 2B, and the binding energies of 368.25 eV for Ag 3d_5/2_ and 374.20 eV for Ag 3d_3/2_ could be attributed to Ag (I) and Ag (0) in A-DNA-Ag NCs [45,46], respectively. Therefore, Ag (I) and Ag (0) exist in A-DNA-Ag NCs.

### 2.3. Optimization of Experimental Conditions for L-Histidine Determination

#### 2.3.1. Response of Different DNA-Ag NCs to L-Histidine

Ag NCs protected by different DNA templates can affect L-histidine detection due to the properties of Ag NCs determined by different base sequences and secondary structures [39,40]. The fluorescence of A, B, C, D, E, and F-DNA-Ag NCs with 400 nM Cu^2+^ in the presence of 20 μM L-histidine was determined. SI Figure S6 shows that their fluorescence increased immediately when L-histidine was added; the relative fluorescence intensity (*F*/*F*_0_, *F*_0_ and *F* are the maximum emission intensities of DNA-Ag NCs with 400 nM Cu^2+^ before and after the addition of 20 μM L-histidine) of A-DNA-Ag NCs was enhanced most obviously, and a more than nine-fold fluorescence enhancement could be observed. Therefore, A-DNA-Ag NCs were chosen as the best candidate for subsequent detection experiments.

#### 2.3.2. Determination of the Optimization Concentration of Cu^2+^ and L-Histidine

The fluorescence of A-DNA-Ag NCs can be quenched by Cu^2+^ through energy or electron transfer [38]. The degree of fluorescence quenching of A-DNA-Ag NCs is determined by the concentration of Cu^2+^. The sensitivity of the sensor is influenced if Cu^2+^ concentrations are too high, and the linear range will be narrow if the Cu^2+^ concentration is too low [38]. Therefore, the optimization concentration of Cu^2+^ can be measured. As shown in Figure S7A, as the concentration of Cu^2+^ increases, the fluorescence of A-DNA-Ag NCs gradually decreases, and it plateaus when the Cu^2+^ concentration is 400 nM. Hence, 400 nM was chosen as the Cu^2+^ optimization concentration. Similarly, L-histidine concentrations were also detected. Figure S7B shows the fluorescence intensity of A-DNA-Ag NCs reaches the maximum when the L-histidine concentration is 20 μM.

#### 2.3.3. Determination of Optimization Incubation Time

The reaction times of A-DNA-Ag NCs and 400 nM Cu^2+^, 400 nM Cu^2+^,and 20 μM L-histidine were determined to improve the sensitivity of the proposed sensor. SI Figure S8A,B show that the reaction time is 15 min for A-DNA-Ag NCs and 400 nM Cu^2+^ and 210 min for 400 nM Cu^2+^ and 20 μM L-histidine. Therefore, 15 min and 210 min were selected as the best reaction times for the following related experiments.

#### 2.3.4. Optimization of pH

The sensitive and accurate detection of L-histidine is greatly affected by the pH value of the PBS buffer solution. Thus, the relative fluorescence intensity (*F*/*F*_0_, where *F*_0_ and *F* are the fluorescence intensities of A-DNA-Ag NCs/Cu^2+^ before and after adding 20 μM L-histidine, respectively) of A-DNA-Ag NCs/Cu^2+^ against the pH value was examined. As shown in SI Figure S9, it gradually increased from pH 5 to 7, reached a maximum at pH 7, and declined from pH 7 to 9. Thus, pH 7 was used for all experiments.

### 2.4. Assay of L-Histidine

**Figure 3:**
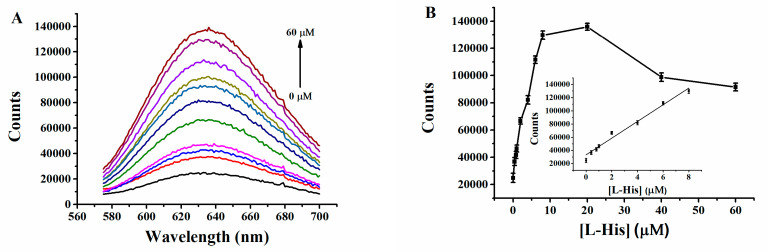
(**A**) Fluorescence emission spectra ofA-DNA-Ag NCs in the presence of L-histidine (0, 0.4, 0.8, 1, 2, 4, 6, 8, 20, 40, and 60 μM). (**B**) L-histidine concentration-dependent changes in fluorescence intensity at 625 nm. Inset: linear between *F*_625nm_ and L-histidine concentration (0–8 μM). Error bars represent the standard deviation of three repetitive experiments.

To validate the performance of the proposed sensor for L-histidine detection, firstly, L-histidine of different concentrations (0–60 μM) was mixed with 400 nM Cu^2+^ in phosphate-buffered solution (20 mM, pH 7.0)and incubated for 210 min to ensure the full reaction of Cu^2+^ and L-histidine. Subsequently, A-DNA-Ag NCs (3 μM) were added into the abovementioned mixture solution and fully reacted for 15 min. Then, the fluorescence of each sample was measured at room temperature. Figure 3A,B show that fluorescence intensity gradually enhanced as L-histidine concentration increased. Due to the high affinity of the imidazole moiety on the L-histidine side chain to Cu^2+^ [38], when L-histidine concentrations were higher, increasing amounts of Cu^2+^ were bound and fewer A-DNA-Ag NCs were quenched; thus, fluorescence intensity was higher. A good linear relationship was observed over the range of 0–8 μM (*R*^2^ = 0.9760, the linear regression equation is *F* = 33,335.0 + 12,647.9*C_L-histidine_*), with a limit of detection (LOD) of 0.096 μM based on 3*σ*_0_/*k* (the value of *σ*_0_ is obtained from the standard deviation of the background, and *k* represents the slope of the calibration line). As shown in SI Table S1, the LOD of the proposed sensor is superior to the previous sensor [22,26,29,32,47,48]. Therefore, the proposed sensor is highly sensitive.

The lifetimes of A-DNA-Ag NCs/Cu^2+^ were studied with an emission wavelength of 625 nm to investigate the fluorescence-quenching mechanism in A-DNA-Ag NCs/Cu^2+^. The fluorescence transients of A-DNA-Ag NCs/Cu^2+^ exhibit exponential haploid time constants (SI Table S2) with an average lifetime of 3.96 ns. The lifetimes of A-DNA-Ag NCs/Cu^2+^ almost do not change with respect to the variation in L-histidine concentration (SI Figure S10). Therefore, this demonstrates that the interaction between A-DNA-Ag NCs/Cu^2+^ and L-histidine is a static process.

### 2.5. Selectivity of the Sensor toward L-Histidine

**Figure 4:**
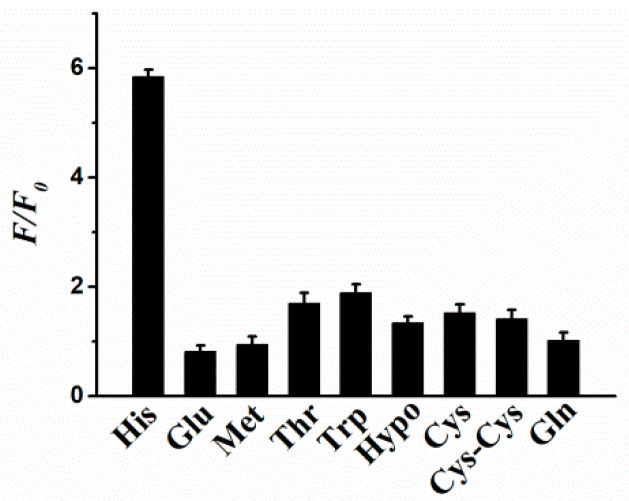
Selectivity experiments on the detection of L-histidine against other amino acids. The relative fluorescence intensity of the fluorescence sensor in the presence of 10 μM of L-histidine and 20 μM of various other amino acids.

A fluorescence sensor with good properties should possess good selectivity because it is an important parameter. Therefore, the relative fluorescence intensity of the proposed sensor was determined using 10 μM of L-histidine and 20 μM of other amino acids. Additionally, in order to eliminate the interference of cysteine and cystine, NEM, as a masking agent, was introduced into the sensor system because the thiol group of cysteine and cystine can interact with Cu^2+^. As shown in Figure 4, the fluorescence response of the sensor to L-histidine was very obvious, while the influence of other amino acids was very weak. On the other hand, The fluorescence response of the proposed sensor towards L-histidine was monitored in the presence of other amino acids. As shown in SI Figure S11, the black bars represent the relative fluorescent intensity (*F*/*F*_0_) of A-DNA-Ag NCs with 400 nM Cu^2+^ in the presence of 10 μM L-histidine, and the red bars represent the *F*/*F*_0_ of A-DNA-Ag NCs with 400 nM Cu^2+^ when 10 μM of L-histidine and 20 μM of another amino acid coexist. The observation shows significant fluorescence enhancements with respect to L-histidine. Although fluorescence also increases a little with respect to other amino acids, it is not very obvious. Thus, the proposed sensor had excellent selectivity.

### 2.6. Detection of L-Histidine in Human Urine

The proposed sensor was employed to detect L-histidine in diluted human urine, and an experiment was carried out to verify its practical application in biological media. In total, 2, 4, 6, and 8 μM of L-histidine were separately mixed with 100-fold diluted human urine samples with 10 mM of NEM added, and detection was carried out utilizing the proposed sensor following the abovementioned method. The results are shown in Table 2. The recoveries of spiked L-histidine are within the range of 98.3% to 105.0% and the RSD values range from 1.81 to 2.93%, thus exhibiting good precision. Therefore, the results illustrate the validity of the sensor for detecting L-histidine in diluted human urine.

## 3. Experimental Section

### 3.1. Materials and Apparatus

Oligonucleotides were provided by Sangon Biotechnology Inc. (Shanghai, China), and the sequences and names of DNA are listed in Table 1. L-histidine (His), L-cystine (Cys-cys), L-glutamine (GLn), L-cysteine (Cys), L-glutamic acid (Glu), L-methionine (Met), L-hydroxyproline (Hypo), L-tryptophan (Trp), L-threonine (Thr), N-ethylmaleimide (NEM), silver nitrate (AgNO_3_, 99.8%), and sodium borohydride (NaBH_4_, 98%) were purchased from Aladdin Bio-chem technology Co., Ltd. (Shanghai, China). Then, a 50 mM NEM solution was prepared in dimethyl sulfoxide (DMSO) and stored in darkness at 4 °C for further use. All chemical reagents were of analytical grade and were used as received without further purification. The phosphate-buffered solution (PBS, 20 mM, pH 7.0) was used in all experiments. All solutions were prepared using Milli-Q water (18.2 MΩ·cm).

A Cary 50 Bio spectrophotometer (Varian Inc., Palo Alto, CA, USA) was used to detect UV–vis absorption spectra at room temperature. Fluorescence spectra were measured using an Edinburgh FS5 fluorescence spectrophotometer (Edinburgh Instruments, Livingston, UK) with excitation slit widths of 2.0 nm and emission at 4.0 nm at ambient temperature. The average size and morphologies of Ag NCs were recorded using a JEOL JEM-2100 transmission electron microscope with an acceleration voltage of 200 kV. Time-resolved fluorescence measurements were performed using an FS5 fluorescent lifetime spectrometer operating in the time-correlated single-photon counting (TCSPC) mode and using a semiconductor laser (405 nm) as the excitation source. Commercial software by Edinburgh Instruments was used for data analyses. When $\sum_{i=1}^{n}A_{i}$ = 1, the average excited state lifetime is expressed by τ_avg_= $\sum_{i=1}^{n}A_{i}\tau_{i}$. The reported spectrum of each sample represents the average of three scans. X-ray photoelectron spectroscopy (XPS) (ESCAL-ab 220i-XL, VG Scientific, Cambridge, UK) was performed using a monochromic Al Ka as a source at 1486.6 eV.

### 3.2. Synthesis of Ag NCs

DNA-Ag NCs were prepared according to the literature [43]. Briefly, 3.0 μM of DNA and 18 μM of Ag NO_3_ solutions were added into PBS solution (pH 7.0, 20 mM). Then, the solution was mixed uniformly using a pipette and kept from light at 4 °C for 20 min. Afterwards,18 μM of freshly prepared NaBH_4_ was promptly added and stirred for 3 min. The abovementioned mixture was incubated in the dark at 4 °C for 1.5 h to obtain DNA-Ag NCs for further use.

### 3.3. Assay of L-Histidine

L-histidine of different concentrations (0–60 μM) was mixed with 400 nM Cu^2+^ in a phosphate-buffered solution (20 mM, pH 7.0), and it was incubated for 210 min. Subsequently, DNA-Ag NCs (3 μM) were added into the abovementioned mixture solution and fully reacted with each other for 15 min. Then, the fluorescence of each sample was measured at ambient temperature.

### 3.4. Application of the Fluorescence Sensor

Urine samples from healthy volunteers were collected and diluted 100-fold. NEM was added to prevent the elimination of the potential effects of biothiols in human urine, and the final concentration of the NEM solution was 10 mM. Then, 2, 4, 6, and 8 μM L-histidine samples were spiked in 100-fold diluted human urine samples containing 10 mM NEM and analysis was carried out utilizing the same method applied in the detection of L-histidine.

### 3.5. Measurement of the Absolute Photoluminescence Quantum Yield

The determination of the absolute photoluminescence quantum yield (APLQY) of this study is based on diffuse reflectance and absorbance spectra, and the SC-30 Integrating Sphere Module from an FS5 Sample Module was used. *η* represents the absolute quantum yield, and its mathematical formula is *η* = *N^em^*/*N^abs^*. In this study, the absolute quantum yield was calculated via direct excitation measurements, where one records the scatter and emission of a sample that is directly excited via radiation from the excitation monochromator alone. 

## 4. Conclusions

To summarize, a label-free, turn-on, simple, cost-effective, and low-toxicity fluorescence sensor was constructed based on Cu^2+^-mediated A-DNA-Ag NCs. The proposed sensor displayed high selectivity and excellent sensitivity. Moreover, the sensor succeeded in detecting L-histidine in diluted human urine. Therefore, its potential prospects for utilization in clinical diagnosis and biological systems are apparent.

## Figures and Tables

**Table 1:** Names and sequences of DNA.

DNA	Sequences (5′–3′)
A-DNA B-DNA C-DNA D-DNA E-DNA F-DNA	CCCCCCCCCCCC CCCTTAATCCCC CCCTAACTCCCC CCTCCTTCCTCC CCCTCTTAACCC CCCTTTAACCCC

**Table 2:** The concentration of L-histidine measured in 100-fold diluted human urine solution (*n* = 3).

Samples	Spiked (μM)	Measured (μM) Means ^a^ ± SD ^b^	Average Recovery (%)	RSD (%)
1	2	2.1 ± 0.05	105.0	2.38
2	4	4.1 ± 0.12	102.5	2.93
3	6	5.9 ± 0.13	98.3	2.17
4	8	8.3 ± 0.15	103.8	1.81

^a^ The mean of three determinations. ^b^ Standard deviation. RSD = relative standard deviation.

## Data Availability

Data are contained within the article and Supplementary Materials.

## Supplementary Materials

The following supporting information can be downloaded at:https://www.mdpi.com/article/10.3390/molecules29102167/s1, Table S1: Comparison of different strategies for the detection of L-histidine; Table S2: The lifetimes of A-DNA-Ag NCs in the absence and presence of different concentrations of L-histidine; Figure S1: The feasibility of the sensor. The fluorescent emission spectra of A-DNA-Ag NCs alone (curve a), (curve b) in the presence of 400 nM Cu^2+^, and (curve c) upon adding 10 mM L-histidine; Figure S2: The excitation (curve a) and emission (curve b) spectra (A, B, C, D, E) of B, C, D, E, and F-DNA-Ag NCs; Figure S3: UV–Vis absorption spectra of A-DNA-Ag NCs under different concentrations of L-histidine; Figure S4: The change in fluorescence intensity of A, B, C, D, E, and F-DNA-Ag NCs with increasing time. Error bars represent the standard deviation of three independent measurements. *c*(DNA) = 3.0 μM; Figure S5: The absolute photoluminescence quantum yield (APLQY) of A, B, C, D, E, and F-DNA-Ag NCs; Figure S6: The relative fluorescence intensity (*F*/*F*_0_) of different DNA-Ag NCs (A, B, C, D, E, and F represent A, B, C, D, E, and F-Ag NCs). *F*_0_ and *F* are the maximum emission intensity of the DNA-Ag NCs with 400 nM Cu^2+^ before and after the addition of 20 mM L-histidine, respectively. The error bars represent the standard deviation of three independent measurements; Figure S7: The fluorescence intensity of A-DNA-Ag NCs as a function of concentration of Cu^2+^ (A) and L-histidine (B). The error bars represent the standard deviation of three independent measurements; Figure S8: The fluorescence intensity of A-DNA-Ag NCs as a function of incubation time of A-DNA-Ag NCs and Cu^2+^ (A), and incubation time of Cu^2+^ and L-histidine (B). The error bars represent the standard deviation of three independent measurements; Figure S9: The relative fluorescence intensity (*F*/*F*_0_) of A-DNA-Ag NCs at the different pH values. *F*_0_ and *F* are the maximum emission intensity of A-DNA-Ag NCs/Cu^2+^ before and after adding 20 μM L-histidine, respectively. The error bars represent the standard deviation of three independent measurements; Figure S10: The fluorescence lifetimes of A-DNA-Ag NCs (excitation at 405 nm and emission at 625 nm) incubated without and with the different concentrations of L-histidine; Figure S11: Selectivity of the L-histidine detection system. The relative fluorescent intensity (*F*/*F*_0_) of A-DNA-Ag NCs with 400 nM of Cu^2+^ in the presence of 10 μM of L-histidine (black bars) and coexistence (red bars) of L-histidine (10 μM) and various other amino acids (20 μM).
