# Supplementary material for: Fluorescence Sensors for the Detection of L-Histidine Based on Silver Nanoclusters Modulated by Copper Ions
Source: Molecules. 2024 May 7;29(10):2167. doi: 10.3390/molecules29102167 (PMC11123781; doi:10.3390/molecules29102167)
Supplement: Supplementary file 1 [file molecules-29-02167-s001.zip › molecules-2934793-supplementary.pdf]

Supplementary Information

Fluorescence Sensors for the Detection of L-histidine Based on Silver Nanoclusters Modulated by Copper Ions

Yuxia Li, Min Li, Liuzhi Hu, Baozhu Zhang*

Department of Chemistry and Chemical Engineering, Jinzhong University, Jinzhong 030619, China

* Correspondence: zhangbaozhu518@126.com

Table of contents

Table S1 Comparison of different strategies for the detection of L-histidine. S3

Table S2 The lifetimes of A-DNA-Ag NCs in the absence and presence of different concentrations of L-histidine. S3

Figure S1. The feasibility of the sensor. The fluorescent emission spectra of A-DNA-Ag NCs alone (curve a), (curve b) in the presence of 400 nM of Cu^{2+}, and (curve c) upon adding 10 mM of L-histidine. S3

Figure S2. The excitation (curve a) and emission (curve b) spectra (A, B, C, D, and E) of B, C, D, E, and F-DNA-AgNCs. S4

Figure S3. UV-Vis absorption spectra of A-DNA-Ag NCs under the different concentrations of L-histidine. S4

Figure S4. The change in fluorescence intensity of A, B, C, D, E, and F-DNA-Ag NCs with increasing time. Error bars represent the standard deviation of three independent measurements. *c*(DNA) = 3.0 μM. S5

Figure S5. The absolute photoluminescence quantum yield (APLQY) of A, B, C, D, E, and F-DNA-Ag NCs. S6

Figure S6. The relative fluorescence intensity (F/F_0) of different DNA-Ag NCs (A, B, C, D, E, and F represent A, B, C, D, E, and F-Ag NCs). F_0 and F are the maximum emission intensity of the DNA-Ag NCs with 400 nM of Cu^{2+} before and after the addition of 20 mM of L-histidine, respectively. The error bars represent the standard deviation of three independent measurements. S6

Figure S7. The fluorescence intensity of A-DNA-Ag NCs as a function of concentration of Cu^{2+} (A) and L-histidine (B). The error bars represent the standard deviation of three independent measurements. S7

Figure S8. The fluorescence intensity of A-DNA-Ag NCs as a function of incubation time of A-DNA-Ag NCs and Cu^{2+} (A), and incubation time of Cu^{2+} and L-histidine (B). The error bars represent the standard deviation of three independent measurements. S7

Figure S9. The relative fluorescence intensity (F/F_0) of A-DNA-Ag NCs at different pH values. F_0 and F are the maximum emission intensity of A-DNA-Ag NCs/Cu^{2+} before and after adding 20 μM of L-histidine, respectively. The error bars represent the standard deviation of three independent measurements. S7

Figure S10. The fluorescence lifetimes of A-DNA-Ag NCs (excitation at 405 nm and emission at 625 nm) incubated without and with the different concentrations of L-histidine. S8

Figure S11. Selectivity of the L-histidine detection system. The relative fluorescent intensity (F/F_0) of A-DNA-Ag NCs with 400 nM of Cu^{2+} in the presence of 10 μM of L-histidine (black bars) and coexistence (red bars) of L-histidine (10 μM) and other various other amino acids (20

μM).

Table S1 Comparison of different strategies for the detection of L-histidine.

Recognition probe	Signal output	LOD	Linear range	References
Ni^{2+}-modulated Hcy-capped CdTe QDs	Fluorescent	0.3 μM	1-30 μM	47
G-quadruplex-based switch-on assay	Luminescent	1.0 μM	1-500 μM	32
L-histidine dependent DNAzymes	Colorimetric	50 μM	50-100 μM	48
Vitamin B6 derivative	Fluorescent	0.3 μM	Not mentioned	26
Indicator-displacement assay	Colorimetric	0.4 μM	2-30 μM	29
EPANS-based probe	Fluorescent	1.0 μM	4-500 μM	22
Fluorescence sensor based on Ag NCs	Fluorescent	0.096 μM	0-8 μM	this work

Table S2 The lifetimes of A-DNA-Ag NCs in the absence and presence of different concentration of L-histidine.

Samples	[L-histidine] (μM)	τ (ns)	χ^2
A-DNA-Ag NCs + 400 nM Cu^{2+}	0	3.98	1.270
	2	3.92	1.247
	4	3.97	1.253
	6	3.94	1.289
	8	3.97	1.302

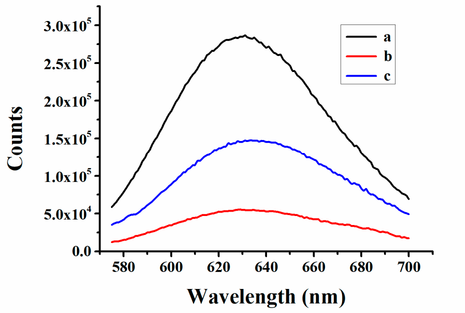

Figure S1. The feasibility of the sensor. The fluorescent emission spectra of A-DNA-Ag NCs alone (curve a), (curve b) in the presence of 400 nM of Cu^{2+}, and (curve c) upon adding 10 mM of L-histidine.

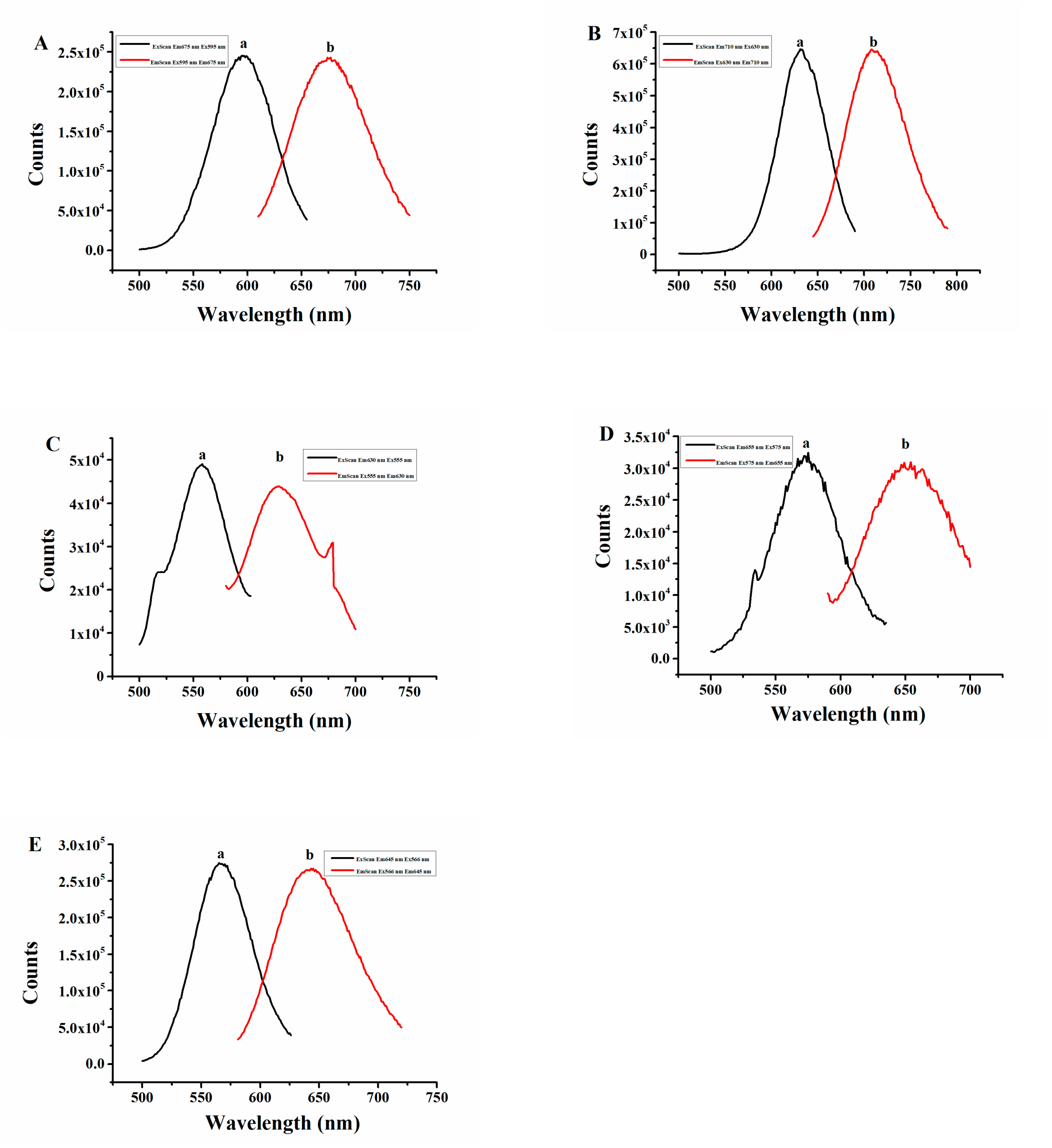

Figure S2. The excitation (curve a) and emission (curve b) spectra (A, B, C, D, and E) of B, C, D, E, and F-DNA-Ag NCs.

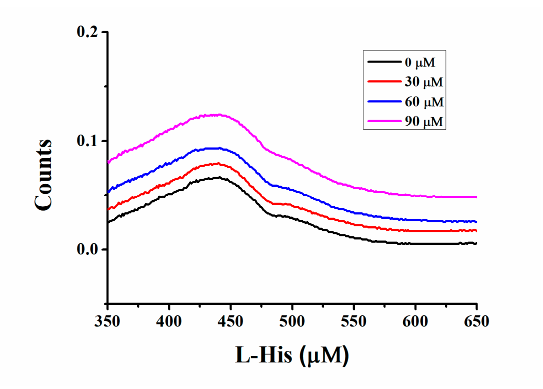

Figure S3. UV-Vis absorption spectra of A-DNA-Ag NCs under the different concentrations of L-histidine.

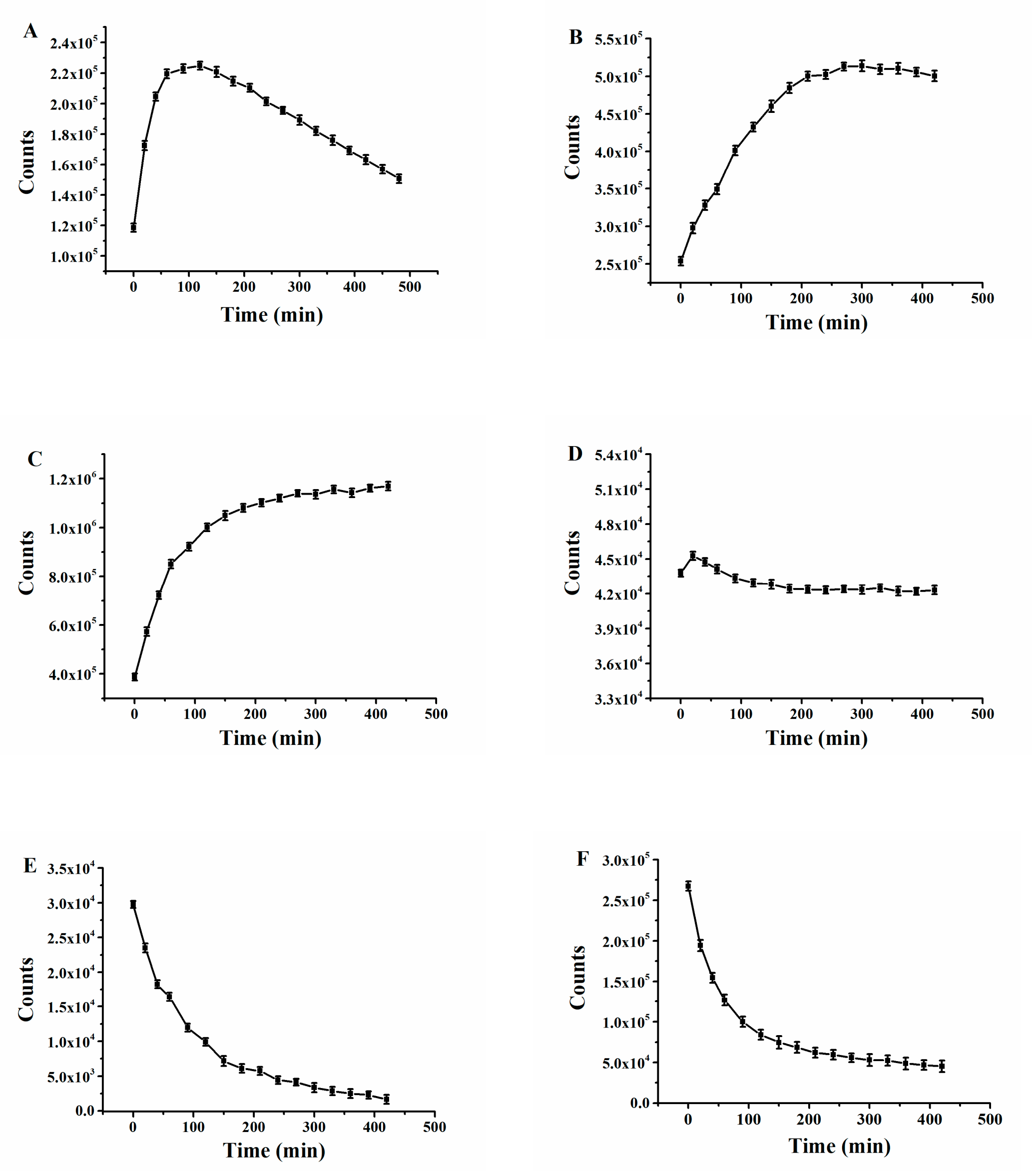

Figure S4. The change in fluorescence intensity of A, B, C, D, E, and F-DNA-Ag NCs with increasing time. Error bars represent the standard deviation of three independent measurements. c(DNA) = 3.0 μM.

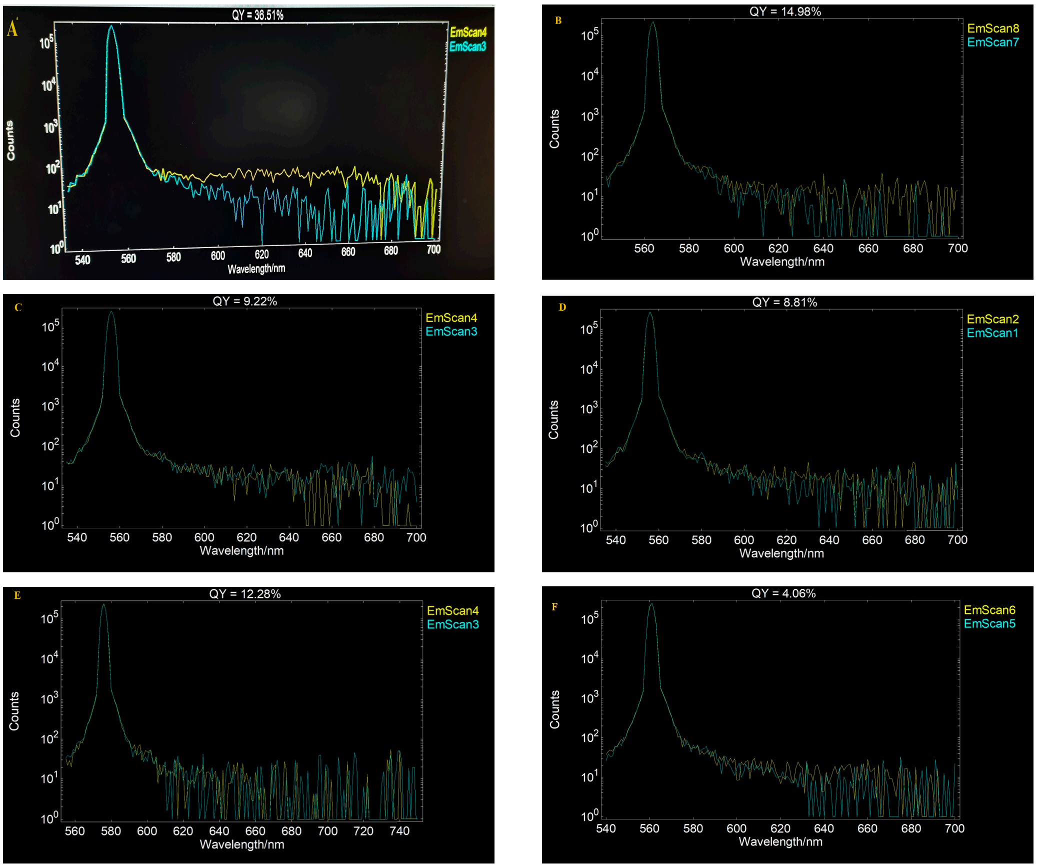

Figure S5. The absolute photoluminescence quantum yield (APLQY) of A, B, C, D, E, and F-DNA-Ag NCs.

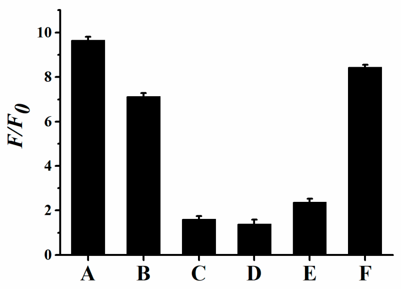

Figure S6. The relative fluorescence intensity (F/F_0) of different DNA-Ag NCs (A, B, C, D, E, and F represent A, B, C, D, E, and F-Ag NCs). F_0 and F are the maximum emission intensity of the DNA-Ag NCs with 400 nM of Cu^{2+} before and after the addition of 20 mM of L-histidine, respectively. The error bars represent the standard deviation of three independent measurements.

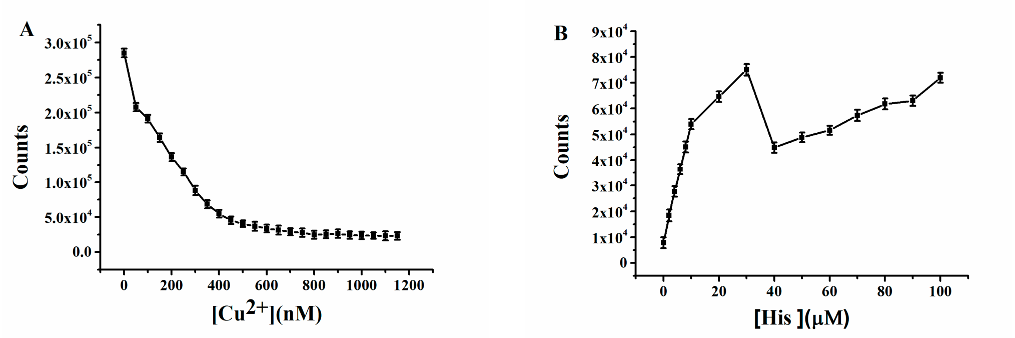

Figure S7. The fluorescence intensity of A-DNA-Ag NCs as a function of concentration of Cu^{2+} (A) and L-histidine (B). The error bars represent the standard deviation of three independent measurements.

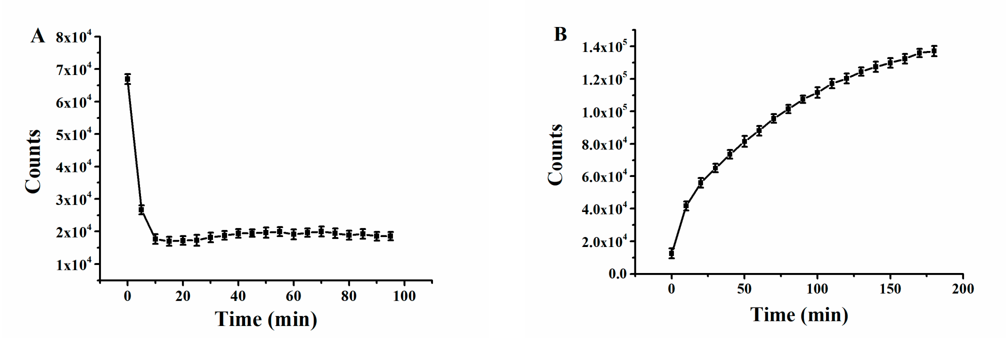

Figure S8. The fluorescence intensity of A-DNA-Ag NCs as a function of incubation time of A-DNA-Ag NCs and Cu^{2+} (A), and incubation time of Cu^{2+} and L-histidine (B). The error bars represent the standard deviation of three independent measurements.

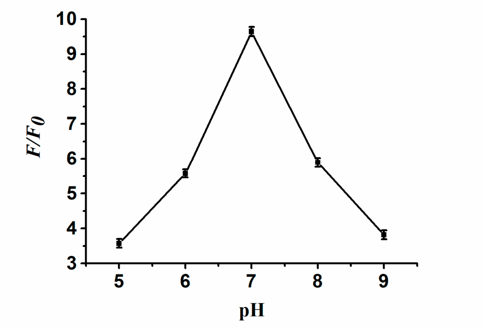

Figure S9. The relative fluorescence intensity (F/F_0) of A-DNA-Ag NCs at different pH values. F_0 and F are the maximum emission intensity of A-DNA-Ag NCs/Cu^{2+} before and after adding 20 μM of L-histidine, respectively. The error bars represent the standard deviation of three independent measurements.

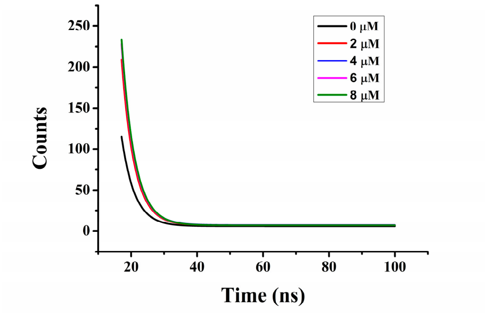

Figure S10. The fluorescence lifetimes of A-DNA-Ag NCs (excitation at 405 nm and emission at 625 nm) incubated without and with the different concentration of L-histidine.

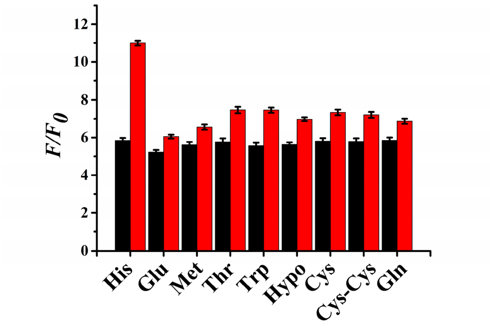

Figure S11. Selectivity of the L-histidine detection system. The relative fluorescent intensity (F/F_0) of A-DNA-Ag NCs with 400 nM of Cu^{2+} in the presence of 10 μM of L-histidine (black bars) and coexistence (red bars) of L-histidine (10 μM) and various other amino acids (20 μM).
